# Prediction of Visual Acuity in Pathologic Myopia with Myopic Choroidal Neovascularization Treated with Anti-Vascular Endothelial Growth Factor Using a Deep Neural Network Based on Optical Coherence Tomography Images

**DOI:** 10.3390/biomedicines11082238

**Published:** 2023-08-09

**Authors:** Migyeong Yang, Jinyoung Han, Ji In Park, Joon Seo Hwang, Jeong Mo Han, Jeewoo Yoon, Seong Choi, Gyudeok Hwang, Daniel Duck-Jin Hwang

**Affiliations:** 1 Department of Applied Artificial Intelligence, Sungkyunkwan University, Seoul 03603, Republic of Korea; mgyang@g.skku.edu (M.Y.); jinyounghan@skku.edu (J.H.); jeewooyoon@raondata.ai (J.Y.); seongchoi@raondata.ai (S.C.); 2 Department of Human-Artificial Intelligence Interaction, Sungkyunkwan University, Seoul 03603, Republic of Korea; 3 Department of Medicine, Kangwon National University Hospital, Kangwon National University School of Medicine, Chuncheon 24341, Gangwon-do, Republic of Korea; parkjiin@gmail.com; 4 Seoul Plus Eye Clinic, Seoul 01751, Republic of Korea; poppn78@daum.net; 5 Seoul Bombit Eye Clinic, Sejong 30127, Republic of Korea; joehan712@gmail.com; 6 RAONDATA, Seoul 04615, Republic of Korea; 7 Department of Ophthalmology, Hangil Eye Hospital, Incheon 21388, Republic of Korea; mehwang78@naver.com; 8 Department of Ophthalmology, Catholic Kwandong University College of Medicine, Incheon 22711, Republic of Korea

**Keywords:** myopic choroidal neovascularization, optical coherence tomography, visual acuity, anti-vascular endothelial growth factor

## Abstract

Myopic choroidal neovascularization (mCNV) is a common cause of vision loss in patients with pathological myopia. However, predicting the visual prognosis of patients with mCNV remains challenging. This study aimed to develop an artificial intelligence (AI) model to predict visual acuity (VA) in patients with mCNV. This study included 279 patients with mCNV at baseline; patient data were collected, including optical coherence tomography (OCT) images, VA, and demographic information. Two models were developed: one comprising horizontal/vertical OCT images (H/V cuts) and the second comprising 25 volume scan images. The coefficient of determination (R^2^) and root mean square error (RMSE) were computed to evaluate the performance of the trained network. The models achieved high performance in predicting VA after 1 (R^2^ = 0.911, RMSE = 0.151), 2 (R^2^ = 0.894, RMSE = 0.254), and 3 (R^2^ = 0.891, RMSE = 0.227) years. Using multiple-volume scanning, OCT images enhanced the performance of the models relative to using only H/V cuts. This study proposes AI models to predict VA in patients with mCNV. The models achieved high performance by incorporating the baseline VA, OCT images, and post-injection data. This model could assist in predicting the visual prognosis and evaluating treatment outcomes in patients with mCNV undergoing intravitreal anti-vascular endothelial growth factor therapy.

## 1. Introduction 

Myopic choroidal neovascularization (mCNV) is a major vision-threatening complication of pathologic myopia and one of the most frequent causes of central vision loss [1,2]. Numerous therapeutic strategies for myopic CNV have been investigated, including focal thermal laser photocoagulation, verteporfin photodynamic therapy, surgical intervention, and intravitreal anti-vascular endothelial growth factor (VEGF) therapy [2]. Of these, optical coherence tomography (OCT) has emerged as the primary medical intervention for mCNV, providing invaluable insights for clinicians to gauge treatment responses and monitor patients [3,4,5].

Several baseline OCT biomarkers, such as mCNV size and location, outer retina integrity, and subfoveal choroidal thickness, have been reported to be associated with visual prognosis [6,7,8,9]; however, an inconsistent consensus remains in the prediction of visual prognosis, while literature analyzing biomarkers for visual acuity (VA) prediction is lacking; therefore, new imaging biomarkers in mCNV require additional study [2,4]. 

With the advancement of artificial intelligence (AI), several research findings have been reported regarding the diagnosis and screening of retinal diseases, including pathologic myopia [10,11,12,13,14]. However, to the best of our knowledge, no attempt has been made to use AI to predict the VA of patients with mCNV undergoing antiangiogenic therapy. Thus, we aimed to develop an AI model to predict the VA of patients receiving intravitreal anti-VEGF injections for mCNV using OCT images prior to anti-VEGF therapy. We also investigated whether the predictive performance of the model could be improved using information (e.g., VA, OCT images, and injection number) after intravitreal anti-VEGF treatment, as well as preinjection information. In addition, we compared the performance of the model achieved using only horizontal/vertical (H/V) OCT images with that achieved using multiple OCT images.

## 2. Materials and Methods

### 2.1. Ethics Statement

This study followed the principles outlined in the Declaration of Helsinki. The research protocol was approved by the Ethics Committee of Hangil Eye Hospital (IRB-21018). Owing to the retrospective observational design of the study, the committee waived the requirement to obtain informed consent.

### 2.2. Data Collection and Labelling 

We analyzed the records of patients who sought treatment at Hangil Eye Hospital between July 2014 and June 2021. MCNV was identified using color fundus photography, fluorescein angiography (FA), and spectral-domain OCT (SD-OCT) (Heidelberg Spectralis, Heidelberg Engineering, Heidelberg, Germany). Patients received 0.5 mg/0.05 mL intravitreal bevacizumab injections (Avastin; Genentech Inc., South San Francisco, CA, USA) using a 30-gauge needle through the pars plana. After the initial injection, an intravitreal injection was administered based on the PRN regimen according to the doctor’s judgment. Patients eligible for the study were diagnosed with mCNV, with foveal subretinal hyperreflective material (SHRM) visible on OCT. The inclusion criteria for mCNV were as follows: high myopia with an axial length greater than 26.0 mm; evident myopic retinal pathological changes like posterior staphyloma, chorioretinal atrophy, papillary crescent, and lacquer cracks; FA revealing active CNV in the subfoveal region; and newly identified CNV cases. The study ruled out patients with age-related macular degeneration (AMD), a history of ocular trauma or surgery, previous subfoveal or juxtafoveal laser treatments, hereditary eye diseases, or any other secondary causes of CNV. Independent retinal specialists diagnosed all mCNV cases using color fundus photography, FA, and OCT. Among these, we used SD-OCT images of patients with mCNV as the data.

We first divided the SD-OCT dataset into two types: H/V cuts and volume scan images. H/V cuts contained a pair of H/V SD-OCT scans, and the volume scan images included 25 volume scan image cuts. Subsequently, we divided them again based on the presence or absence of data 1, 2, and 3 years after the baseline point, as we aimed to predict the VA at these time points. If no data were available at particular and preceding time points, the patient was not included in the corresponding prediction dataset. We also excluded cases in which intraocular surgery was performed within the predicted timeframe from baseline. The six constructed SD-OCT datasets are listed in Table 1.

In addition to the SD-OCT datasets, demographic information of the patients, such as age, sex, and best-corrected VA, was collected. Best-corrected VA was converted to the logarithm of the minimum angle of resolution (logMAR). As the treatment for mCNV is intravitreal bevacizumab injection, we also concentrated on the number of injection treatments performed annually. Table 2 shows the data lists used for VA prediction 1, 2, and 3 years after the baseline point.

### 2.3. Data Preprocessing

To utilize each SD-OCT dataset, we initially downsampled the images to a size of 120 × 120 RGB channels. This was necessary because the deep neural network only accepts images of a fixed size. To prevent overfitting, we performed data augmentation to develop a robust model that can accommodate various input images. This process included random horizontal image flips and random rotations of up to 15°. Data augmentation was performed only during the training phase. 

### 2.4. Model Architecture

Two models were developed to predict VA after N years (N = 1, 2, and 3) from baseline. One model used H/V cuts, and the other model used volume scan images as inputs. VGG-19 [15] was chosen as the base feature extractor instead of other CNN architectures like Resnet-50 [16] and Inception-V3 [17] because it performed better. Transfer learning was applied to prevent overfitting and expedite model training [18] by initializing the CNN layers with pretrained weights from VGG-19 obtained from a large-scale ImageNet dataset [19].

**Figure 1:**
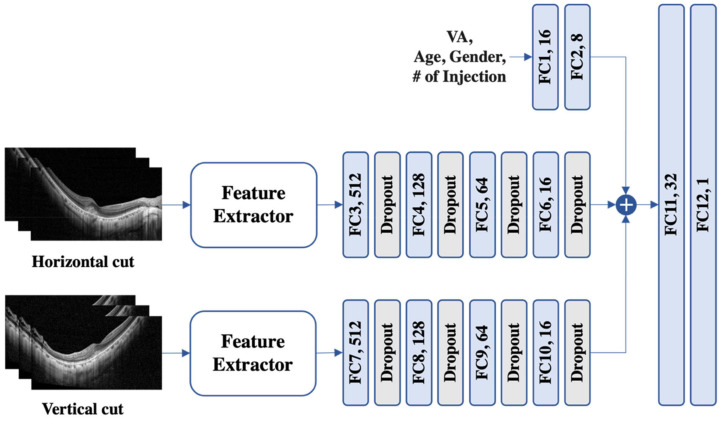
An illustration of the proposed model for horizontal and vertical images. To process variables such as visual acuity (VA), age, sex, and the number (#) of injections, a set of two fully connected (FC) layers was used. The horizontal and vertical cuts were subjected to feature extraction and subsequently traversed a sequence of four FC layers supplemented with dropout. Then, the three extracted features were concatenated and fed into another pair of FC layers to predict VA.

The model for H/V cuts (see Figure 1) uses a feature extractor, followed by four fully connected layers and dropout layers with a leaky ReLU [20] as the activation function. VA, age, sex, and the number of injections were also passed through two fully connected layers before being concatenated with the features from the H/V cuts. Finally, the concatenated feature was fed into two fully connected layers to predict the VA.

**Figure 2:**
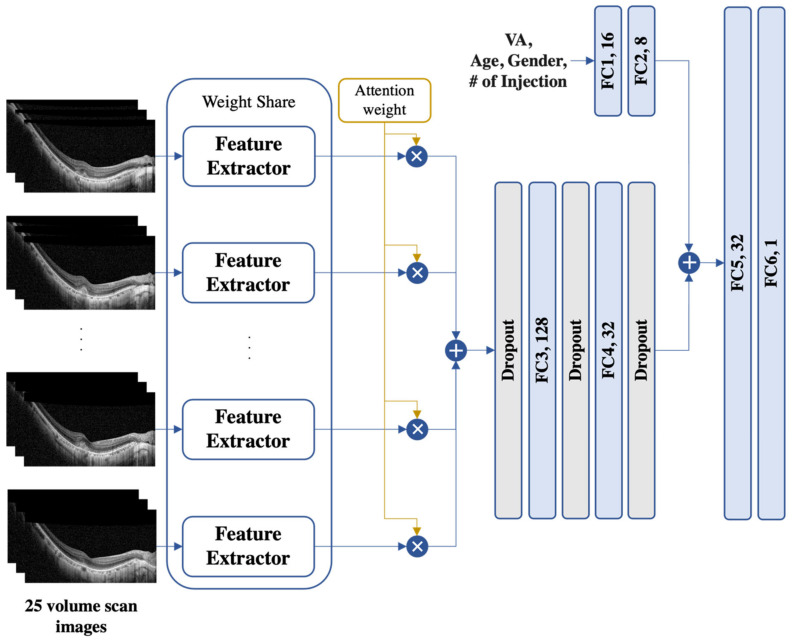
An illustration of the proposed model for 25 volume scan images. The proposed model is a multi-instance model with an attention module to handle multiple SD-OCT images. The fused features were processed through dropout and fully connected (FC) layers, while the demographic information (i.e., age and the number (#) of injections) was processed with a set of two FC layers separately. The resulting features were then concatenated and passed through two FC layers for VA prediction. SD-OCT, spectral-domain optical coherence tomography; VA, visual acuity.

For the volume scan image model (see Figure 2), a multi-instance model structure was adopted to consider multiple SD-OCT images simultaneously. An attention module [21] was used to combine all the volume scan image information by calculating the attention score of each image. The fused feature was then passed through three dropout and two fully connected layers using a leaky ReLU [20] as the activation function. Similarly, VA, age, sex, and the number of injections were passed through two fully connected layers. Finally, all features were concatenated and passed through two fully connected layers to predict the VA.

### 2.5. Experiment Setup 

We assessed our models through two distinct tasks: (i) comparing the two models proposed in this study and (ii) contrasting our model with existing models.

In the former task, we conducted three experiments to predict the VA at 1, 2, and 3 years from baseline. Therefore, we constructed six datasets depending on the type of SD-OCT image and the predicted year. These datasets were divided into training, validation, and test sets in a 70:10:20 ratio, with patient stratification to ensure each patient appeared in only one set. To ensure a fair comparison between the model architectures, we trained them using the same parameters, except for the batch size. The models were trained for 100 epochs with a learning rate of 0.0001 using the mean squared error (MSE) as the loss function with Adam optimization [22]. To prevent overfitting, we used a dropout rate of 0.5 and early stopping with a patience value of 5. We set the batch size to 4 and 64 for the models using volume scan images and H/V cuts, respectively, because the models for volume scan images process multiple images simultaneously, which incurs a higher computational cost.

In the latter task, we compared our model (the volume scan image model) with existing models. Among them, none handled 25 volume scan images and meta-information (i.e., age, number of injection) simultaneously to predict VA. Additionally, no studies have processed multiple time-point volume scan images together for prediction at 1–3 year intervals. Consequently, we performed comparative experiments with research that utilized 25 or more volume scan images together and studies that solely relied on metainformation for 1-year VA prediction. The compared models are summarized as follows:

- ResNet-50 v2 [23]: ResNet-50 v2 CNN architecture [24]. Afterward, it passes through the final dense layer;
- LassoCV [25]: Linear regression method with an L1-norm penalty. It trains the weights to be close to zero, thereby identifying the most important features in the model and finding a generalized model;
- LR + RF [26]: Combination of LR and RF into an ensemble algorithm using a stacking approach. LR refers to linear regression, and RF stands for random forest regressor.

For a fair comparison, our model only utilized baseline OCT and VA, along with injection data from the first year, without considering multiple time points (i.e., next visit). 

## 3. Results

We conducted a study based on OCT images from 279 Korean patients with 8444 H/V OCT images and 107,975 volume scan images 1 year after the initial anti-VEGF injection. Thereafter, in the second and third years, fewer images were used, the details of which are presented in Table 1. Among the 279 patients, 65 were male (23.3%), and the mean age was 50.85 ± 15.89 years at baseline. The VA values at baseline and after 1, 2, and 3 years were 0.48 (0.33), 0.47 (0.34), 0.51 (0.31), and 0.56 (0.27) logMAR (decimal BCVA), respectively. No significant improvement in the VA was observed in the first year (*p* = 0.156); however, a significant change was observed in the second (*p* = 0.005) and third years (*p* = 0.021). The average number of injections was 1.06 ± 0.83 in the first year, 1.30 ± 1.20 in the second year, and 1.63 ± 1.54 in the third year.

### 3.1. Prediction 1 Year from the Baseline

Our study found that baseline SD-OCT images alone were not very effective in predicting VA 1 year after baseline, with low R^2^ accuracy of −0.009 (RMSE = 0.503) and 0.016 (RMSE = 0.471) for H/V cuts and volume scan images, respectively (Table 3). On the other hand, baseline VA alone led to a higher performance, but the models that included both baseline SD-OCT images and VA had better R^2^ accuracy (R^2^ = 0.849 (RMSE = 0.194) and 0.854 (RMSE = 0.194), respectively). While demographic information and the number of injections in the first year were not helpful, SD-OCT images and VA at the next visit markedly increased the prediction performance. The next visit refers to the first followup visit after the injection, which usually occurs within 1–2 month. The models achieved the highest performance when using SD-OCT images and VA at the baseline and next visits (R^2^ = 0.905 (RMSE, 0.154) and 0.911 (RMSE = 0.151), respectively). 

### 3.2. Prediction 2 Years from the Baseline

Table 4 demonstrates that using either baseline SD-OCT images or VA alone had a low ability to predict VA 2 years after baseline, as indicated by the low R^2^ values for both H/V cuts and volume scan images. However, including both baseline SD-OCT images and VA in the models achieved better R^2^ accuracy (R^2^ = 0.729 (RMSE = 0.374) and 0.753 (RMSE = 0.336), respectively). Furthermore, when SD-OCT images and VA 1 year after baseline were added, the model showed higher performance (R^2^ = 0.725 (RMSE = 0.373) and 0.809 (RMSE = 0.339), respectively). The highest accuracy was achieved by utilizing all available data, except demographic information (R^2^ = 0.839 (RMSE = 0.285) and 0.894 (RMSE = 0.254), respectively). Interestingly, the number of injections administered in 1 and 2 years was helpful in prediction. Notably, the number of injections in the first year was observed to have a positive impact on performance only when used in conjunction with VA at the 1-year prediction time point (R^2^ = 0.813 (RMSE = 0.308) and 0.877 (RMSE = 0.272), respectively) and not otherwise (R^2^ = 0.662 (RMSE = 0.432) and 0.671 (RMSE = 0.426), respectively). This implies that the correlation between the number of injections in the first year and VA 1 year after the baseline contributed to the successful prediction of VA after 2 years.

### 3.3. Prediction 3 Years from the Baseline

As shown in Table 5, predicting VA 3 years after the baseline was difficult using only baseline SD-OCT images and VA. Even with their combined use, the R^2^ value remained at 0.579 (RMSE = 0.450) and 0.602 (RMSE = 0.433) for the H/V cuts and volume scan images, respectively. However, the number of injections in the N-th year (N = 1, 2, and 3) and the SD-OCT images and VA after the M-th year (M = 1 and 2) from the baseline significantly contributed to performance improvement. The highest accuracy was achieved by incorporating all available data except demographic information, with R^2^ values of 0.818 (RMSE = 0.296) and 0.891 (RMSE = 0.227). This indicates that the events that occurred each year (i.e., injection) and the SD-OCT and VA information at each time point are important predictors of VA 3 years after baseline.

Overall, R^2^ and RMSE were better when using all 25 volume scan images than when using only H/V cuts. Among the baseline VA and OCT data, baseline VA was the most important predictor of VA after 1, 2, and 3 years. The baseline volume scan OCT information helped predict VA at 1, 2, and 3 years, whereas the baseline H/V cut OCT information was helpful only in predicting VA at 1 and 2 years but offered no significant help in predicting VA at 3 years. As the prediction time increased from 1 to 3 years, the contribution of additional information, such as SD-OCT images and the number of injections, increased, but the overall performance decreased. 

### 3.4. Comparison with Existing Models

As shown in Table 6, our model outperformed other existing models. Specifically, relative to ResNet-50 v2 [23], the performance was notably poor (R^2^ = −0.130 (RMSE = 0.555)). This indicates the importance of the attention module, as the performance of our model that relying solely on SD-OCT images was higher. Moreover, with regards to meta-information (VA and number of injections), previous models [25,26] exhibited approximately 4–6% lower performance (R^2^ = 0.795 (RMSE = 0.237) and 0.765 (RMSE = 0.253), respectively) compared to our model. This underscores the significance of combining volume scan images and metainformation for accurate VA prediction.

## 4. Discussion

In our study involving patients with mCNV undergoing antiangiogenic therapy, we were able to predict VA 1 year later with more than 80% accuracy using only baseline VA and OCT images prior to injection. Furthermore, when post-injection VA and OCT followup data were also provided, we demonstrated the ability of our model to predict VA a year later with over 90% accuracy. While functional information such as VA was of utmost importance in predicting VA, OCT images also contributed to further improvements in predictive performance. Additionally, compared with using only H/V cuts, we found that using multiple volume scan OCT images enhanced our predictive accuracy.

Research on the prediction of VA in patients with mCNV is scarcely found [27]. According to a recent study by Inoda et al. [27], prediction of VA in mCNV was inferior to that in neovascular age-related macular degeneration (nAMD) or retinal vein occlusion, with their estimated and actual VA corresponding to an R^2^ value of 0.36. However, their study aimed to predict VA using cross-sectional OCT images at a single time point rather than predicting post-injection VA with preinjection OCT images. In addition, the number of patients with mCNVs was small (N = 13), making it difficult to directly compare the results of our study. This discrepancy may also arise from differences in the study cohorts and designs. Studies predicting VA post injection have primarily been conducted in nAMD. Dun Jack Fu et al. [28] demonstrated approximately 40% (R^2^ = 0.378) predictive accuracy for VA at 1 year using only baseline OCT and VA. They used quantitative OCT biomarker information, unlike our study; however, similar to our findings, VA information was a more important factor contributing to VA prediction than OCT imaging. In addition, the fact that the predictive power increased further when VA and OCT change information was added is similar to our results. In predicting post-injection VA in patients with mCNV, an increase in predictive performance when functional and anatomical changes are reflected may suggest that the response to the injection treatment itself is a predictive factor for long-term visual outcome progression, which is consistent with the findings of previous studies [28].

In our study, the significance of baseline VA information outweighed that of baseline OCT images in predicting post-injection VA. This arises from the fact that predicting the post-injection VA implies predicting the patient’s functional ability. As a result, the preinjection VA may be more critical than the anatomical configuration reflected in the OCT images. Nevertheless, we confirmed that predictive performance increased when additional OCT information was provided. Based on our results, we believe that OCT images can meaningfully contribute to VA prediction in patients with mCNV in clinical settings using a deep learning model. Future research should identify how and to what extent OCT images without segmentation can be meaningfully combined with VA to increase the overall predictive value. In addition, demographic factors such as sex and age did not provide additional help in predicting VA in years 1, 2, and 3. OCT images may reflect information such as the sex and age of patients. Alternatively, this may be because demographic factors such as age or sex have no special correlation with the injection effect or treatment prognosis of mCNV. It is unclear why these demographic factors did not provide additional help in predicting the VA, necessitating further studies. 

The accuracy of VA prediction decreased from the first to the second and third years. The actual VA was significantly lower than baseline in the second and third years post injection, potentially due to fibrous scar formation, with photoreceptor damage from SHRM over time after the onset of mCNV, leading to a poor anti-VEGF response. Furthermore, as the time to predict VA from baseline increases to 2 and 3 years, the likelihood of events such as mCNV recurrence may increase, which may reduce the VA-predictive power. Although our study did not investigate the recurrence of mCNV and only counted the number of injections, the fact that the average number of injections increased as the followup period increased suggests recurrence during followup. Along with these changes, changes in lens status and functional changes in the optic nerve may have made VA prediction more challenging. However, in the second and third years, the predictive power could be improved by adding the number of previous injections and followup information from OCT and VA. When predicting long-term VA, we learned that OCT images and VA information obtained during followup observation—not just baseline—also helped predict later VA. 

In our study, using 25 volume scan images at 1, 2, and 3 years improved the VA-predictive power compared with using only H/V images. Previous deep learning studies using OCT-based convolutional neural network (CNN) models predicted VA using fewer than five H/V OCT images [27,29]. This implies that training with more OCT images per eye, as in our study, enhances the model’s predictive performance, which requires validation in future studies using OCT-based deep learning models for VA prediction. Studies have predicted VA using quantitative OCT biomarker information obtained through the segmentation of multiple OCT images in patients with nAMD [23,28,30]. Predicting VA in mCNV using quantitative OCT image information would be interesting but is beyond the scope of our current research.

As previously mentioned, incorporating multiple OCT images per eye resulted in improved predictive performance for VA. However, this would not have been possible without the inclusion of an attention module [21]. When the model for the 25 volume scan images was trained without the attention module, the performance notably declined. This disparity can be attributed to the fact that with only two images (horizontal and vertical), the model can easily discern the relevant regions that contribute to the prediction. In contrast, with 25 volume scan images, it is challenging for the model to determine which areas are crucial and informative for accurate predictions. Consequently, when deploying deep learning in various ophthalmology tasks and utilizing multiple OCT images, the incorporation of an attention module becomes imperative to enhance the model performance.

Our study is subject to several limitations. First, we used a relatively small number of images from a single OCT device, and the retrospective nature of our study resulted in fewer patients in the second and third years than in the first year. The difference in the number of patients may affect the importance of factors (e.g., VA and OCT) that are helpful in predicting long-term VA. Also, due to the retrospective nature of the study, the dataset used in our study includes a higher proportion of patients who may not have experienced significant improvement through anti-VEGF therapy or may have experienced relapse or other concomitant conditions, leading them to continue visiting the hospital. Second, our study used only macular OCT images and VA, age, and sex information; we did not perform lens status or optic nerve evaluations. However, we initially intended for our study to determine the extent to which post-injection visual acuity can be predicted using VA and OCT. Third, our study was an OCT-based study using a CNN, and quantitative OCT parameter analysis was not performed; therefore, we did not explore OCT biomarkers affecting VA prediction separately. Fourth, our study has not been validated on other databases, and only Koreans were included in the study. Therefore, further validation studies utilizing other databases, particularly those including other ethnicities, are warranted. Finally, it was difficult to determine the exact correlation between the number of injections and mCNV condition because we did not investigate mCNV exacerbation or recurrence after injection. However, because the PRN regimen was followed, an increase in the number of injections may have been positively correlated with the worsening or recurrence of mCNV.

## 5. Conclusions

Our study proposed the use of deep neural networks to predict long-term VA in patients with mCNV. Using a CNN, we were able to predict VA with more than 50% accuracy for 3 years using only baseline information and more than 80% accuracy when additional followup information was used. Our model can help predict the visual prognosis of patients before injection in future clinical settings or assess the long-term VA prognosis of patients during intravitreal anti-VEGF injection treatment.

## Figures and Tables

**Table 1:** Characteristics of patients in each SD-OCT dataset.

	Horizontal/Vertical Cut Images			Volume Scan Images		
	After 1 Year	After 2 Years	After 3 Years	After 1 Year	After 2 Years	After 3 Years
Images, n	8444	5302	3290	107,975	67,850	42,375
Patients, n	279	192	142	279	192	142

SD-OCT, spectral-domain optical coherence tomography.

**Table 2:** Data lists used for VA prediction after 1, 2, and 3 years from baseline point.

	Data
After 1 year	- VA and SD-OCT images at baseline and next visit - The number of injections in 1 year - Age and sex
After 2 years	- VA and SD-OCT images at baseline and after 1 year - The number of injections in 1 year and 2 years - Age and sex
After 3 years	- VA and SD-OCT images at baseline and after 1 and 2 year(s) - The number of injections in 1, 2, and 3 years - Age and sex

VA, visual acuity; SD-OCT, spectral-domain optical coherence tomography.

**Table 3:** Performance of the proposed models in predicting VA 1 year after the baseline.

	H/V Cut		Volume Scan Image	
	RMSE	R^2^	RMSE	R^2^
OCT at baseline	0.50346	−0.00979	0.47105	0.01619
VA at baseline	0.19883	0.84251	0.19626	0.84937
OV(B) ^a^	0.19448	0.84931	0.19354	0.85352
OV(B) + sex + age	0.22167	0.80785	0.20682	0.82959
OV(B) + Inject(1) ^b^	0.20599	0.83096	0.20234	0.83989
OV(B) + OV(N) ^c^	0.15446	0.90496	0.15069	0.91120
OV(B) + OV(N) + Inject(1)	0.16023	0.89772	0.15662	0.90407

^a^ OV(B): SD-OCT images at baseline + VA at baseline. ^b^ Inject(1): the number of injections in the first year. ^c^ OV(N): SD-OCT images at the next visit + VA at the next visit. SD-OCT, spectral-domain optical coherence tomography; VA, visual acuity.

**Table 4:** Performance of the proposed models in predicting VA 2 years after the baseline.

	H/V Cut		Volume Scan Image	
	RMSE	R^2^	RMSE	R^2^
OCT at baseline	0.72555	−0.03815	0.71943	0.04066
VA at baseline	0.37775	0.71859	0.37080	0.72102
OV(B) ^a^	0.37366	0.72886	0.33639	0.75303
OV(B) + sex + age	0.44571	0.64150	0.41487	0.69102
OV(B) + Inject(1) ^b^	0.43218	0.66193	0.42637	0.67138
OV(B) + OV(1) ^c^	0.37321	0.72532	0.33949	0.80934
OV(B) + OV(1) + Inject(1)	0.30815	0.81273	0.27233	0.87732
OV(B) + OV(1) + Inject(1) + Inject(2) ^d^	0.28549	0.83927	0.25370	0.89353

^a^ OV(B): SD-OCT images at baseline + VA at baseline. ^b^ Inject(1): the number of injections in the first year. ^c^ OV(1): SD-OCT images 1 year after the baseline + VA 1 year after the baseline. ^d^ Inject(2): the number of injections in the second year. SD-OCT, spectral-domain optical coherence tomography; VA, visual acuity.

**Table 5:** Performance of the proposed models in predicting VA 3 years after the baseline.

	H/V Cut		Volume Scan Image	
	RMSE	R^2^	RMSE	R^2^
OCT at baseline	0.71571	−0.06554	0.69769	0.02714
VA at baseline	0.44776	0.57128	0.44478	0.57310
OV(B) ^a^	0.45006	0.57866	0.43333	0.60151
OV(B) + sex + age	0.46143	0.55710	0.45674	0.55581
OV(B) + Inject(1) ^b^	0.47352	0.53359	0.46282	0.54390
OV(B) + OV(1) ^c^	0.39429	0.67661	0.35941	0.72495
OV(B) + OV(1) + Inject(1)	0.36825	0.71792	0.33943	0.75469
OV(B) + OV(1) + OV(2) ^d^	0.32497	0.78032	0.27473	0.83928
OV(B) + OV(1) + OV(2) Inject(1) + Inject(2) ^e^	0.30425	0.80744	0.24435	0.87287
OV(B) + OV(1) + OV(2) Inject(1) + Inject(2) + Inject(3) ^f^	0.29614	0.81758	0.22661	0.89066

^a^ OV(B): SD-OCT images at baseline + VA at baseline. ^b^ Inject(1): the number of injections in the first year. ^c^ OV(1): SD-OCT images 1 year after the baseline + VA 1 year after the baseline. ^d^ OV(2): SD-OCT images 2 years after the baseline + VA 2 years after the baseline. ^e^ Inject(2): the number of injections in the second year. ^f^ Inject(3): the number of injections in the third year. SD-OCT, spectral-domain optical coherence tomography; VA, visual acuity.

**Table 6:** Performance comparison with existing models in predicting VA 1 year after the baseline.

		RMSE	R^2^
O(B) ^a^	ResNet-50 v2 [23]	0.55545	−0.12990
V(B) ^b^ + Inject(1) ^c^	LassoCV [25]	0.23657	0.79503
	LR + RF [26]	0.25328	0.76507
OV(B) ^d^ + Inject(1)	Ours	0.20234	0.83989

^a^ O(B): 25 SD-OCT volume scan images at baseline. ^b^ V(B): VA at baseline. ^c^ Inject(1): the number of injections in the first year. ^d^ OV(B): 25 SD-OCT volume scan images at baseline + VA at baseline.

## Data Availability

Data are not available for public access because of patient privacy concerns but are available from the corresponding author upon reasonable request.
